# Multicolor imaging in central serous chorioretinopathy – a quantitative and qualitative comparison with fundus autofluorescence

**DOI:** 10.1038/s41598-019-48040-4

**Published:** 2019-08-13

**Authors:** Vishal Govindahari, Sumit Randhir Singh, Bindu Rajesh, Roberto Gallego-Pinazo, Rosa Dolz Marco, Dhanya V. Nair, Unni Nair, Jay Chhablani

**Affiliations:** 1 Smt. Kanuri Santhamma Centre for Vitreo-Retinal Diseases, L V Prasad Eye Institute, Hyderabad, India; 2 Ophthalmology and Visual Sciences department, Khoo Teck Puat Hospital(KTPH), 90 Yishun Central, Singapore, Singapore; 3 Unit of Macula, Oftalvist Clinic, Valencia, Spain; 4 Department of Retina, Chaithanya Eye Hospital and Research Institute, Trivandrum, Kerala India

**Keywords:** Retinal diseases, Imaging and sensing

## Abstract

Central serous chorioretinopathy (CSCR) is characterised by choroidal hyperpermeability which results in neurosensory detachments (NSD) along with numerous retinal pigment epithelium (RPE) alterations such as RPE atrophy. Fundus autofluorescence (FAF) demonstrates the functionality of the RPE while multicolor imaging(MCI), by means of its three incident wavelengths, provides insight into clinical changes at various levels of the retina and choroid in CSCR. This study compares various clinical findings in CSCR (NSD, subretinal deposits, RPE atrophy, pigment epithelial detachments (PED) and pachyvessels) on the above mentioned imaging modalities both qualitatively and quantitatively. MCI showed higher mean cumulative area of RPE atrophic patches (6.3 ± 6.02 vs 5.7 ± 5.7 mm^2^, p = 0.046), PED (1.3 ± 1.4 vs 1.1 ± 1.2 mm^2^, p = 0.068) and NSD (17.2 ± 11.4 vs 15.7 ± 10.7 mm^2^, p = 0.033). MCI demonstrated better defined lesions (NSD, PED, RPE atrophy) and more number of eyes with PED and pachyvessels in comparison to FAF.Both investigations had a 100% sensitivity in detecting NSD and 100% specificity for sub retinal deposits. This study demonstrates the ability of MCI to quantitatively and qualitatively define various clinical features in CSCR and the advantages it holds over FAF. MCI can hence be considered as a useful imaging modality in documenting and monitoring various structural changes in eyes with CSCR.

## Introduction

Multicolor imaging (MCI) is a confocal-scanning laser ophthalmoscopy based imaging system, which captures reflectance from three monochromatic laser sources which include blue (488 nm), green (515 nm) and infrared (820 nm) wavelengths. The resultant composite is an amalgamation of blue, green and infra-red reflectance images. The unique feature of multicolor imaging is the differential wavelength penetration and the ensuing reflectance leading to better visualization of structures at different depths. The blue reflectance highlights the vitreoretinal interface and inner retinal lesions such as epiretinal membranes. The green reflectance enhances retinal blood vessels and intraretinal exudation while the infrared reflectance helps better visualization of outer retina including retinal pigment epithelium (RPE) and choroid^1,2^.

Central serous chorioretinopathy (CSCR) is characterized by neurosensory detachments (NSD) along with the presence of a wide spectrum of choroidal, retinal pigment epithelium and retinal changes, which occur over a varied time frame. RPE changes in the form of pigment epithelial detachments (PED), RPE hypertrophy and atrophy have been associated with different stages of the disease^3^. Subretinal deposits, which result from the accumulation and subsequent phagocytosis of photoreceptor outer segments as a result of lack of RPE-outer retinal apposition, is  yet another important clinical sign in the CSCR spectrum^4^. Recently, CSCR has been included in the pachychoroid spectrum, which is defined by the presence of dilated larger choroidal vessels (Haller’s layer) with an eventually increased choroidal thickness and secondary compression of the overlying choriocapillaris and Sattler’s layer^5^.

Fundus autofluorescence (FAF) is an imaging modality, which is driven by the accumulation of fluorophores (molecules which absorb and emit specific wavelengths)^6–8^. The autofluorescence pattern and its evolution has been well described in CSCR based on the clinical status of the RPE and chronicity of the disease^9,10^. A functional correlation with visual acuity of certain specific findings such as hypoautofluorescence secondary to RPE atrophy has made FAF a very useful tool as an *in-vivo* indicator of photoreceptor and RPE health^11–13^. This additional attribute as a functional marker enhances the importance of FAF in CSCR and hence it serves as an ideal imaging model to compare with.

Most of the reviews on CSCR describe a multimodal approach in describing and understanding the disease^14,15^. Spectral domain OCT (SD-OCT) has been used as the primary modality to detect activity and in combination with optical coherence tomography angoiography (OCT-A) to detect choroidal neovascular membranes(CNVM)^3,16^. Angiography depicts leak locations, changes at the RPE and choroidal levels along with CNVM detection and follow-up^17,18^. FAF (short wavelength and near-infrared) have great utility in understanding structural alterations at the level of outer retina and RPE along with their corresponding functional effects^10,12^.Considering the structural changes at the various levels of  neuro-sensory retina, RPE and the choroid, MCI may demonstrate a composite picture of all these changes.

Most of the previous work involving MCI, looked at wide variety of retinal and choroidal conditions, which include epiretinal membranes, subretinal deposits, geographic atrophy, age-related macular degeneration and choroidal nevus^2,19–22^. The aim of our study was to quantify and qualify different clinical findings in CSCR on MCI and compare the same with FAF.

## Methods

This is a retrospective study of patients with a diagnosis of CSCR who presented at two study sites, one in India and the other in Spain. Institutional review board approval from the ethics committee at LV prasad eye institute, Hyderabad was obtained for reviewing the recorded patient data and the study adhered to tenets of declaration of Helsinki. An informed consent was obtained from all subjects involved in the study and all the methods were conducted in accordance to relevant guidelines and regulations.

A diagnosis of CSCR was made based on clinical examination and imaging, which included MCI, FAF and optical coherence tomography (OCT). Presence of single or multiple neurosensory detachments associated with retinal pigment epithelium (RPE) changes, which include RPE detachments, atrophy or hypertrophy and a thickened choroid were the OCT criteria for the diagnosis of CSCR. Presence of previously described hypo or hyperautofluorescence patterns based on chronicity of disease, were considered as the FAF criteria for the diagnosis of CSCR^3^.

Patients who underwent MCI and FAF imaging on the same day and had good quality images were included in the study. Multicolor and FAF images of the posterior pole were acquired using Spectralis (HRA- OCT Spectralis, Heidelberg Germany). Images of 30^0^ field  of the posterior pole (both MCI and FAF) were included for image analysis. The corresponding spectral domain OCT (SD-OCT, HRA-OCT Spectralis, Heidelberg Germany) images were referred to during image analysis.

### Image analysis

1. Characteristics of five different findings were analysed and measured or graded. These included  neurosensory detachment (NSD), subretinal deposits, retinal pigment epithelium (RPE) atrophy, pigment epithelial detachments (PED) and pachyvessels. Imaging features of each finding in a given modality have been described in Table 1.

**Table 1:** Imaging features of five clinical findings in central serous chorioretinopathy(CSCR) on fundus autofluorescence (FAF) and rmulticolor imaging (MCI).

Finding	FAF	MCI
RPE^a^ atrophy	Confluent hypoautofluorescent patches^10^	Areas with enhanced visualization of choroidal vessels (bright orange) in comparison to surrounding unaffected area
PED^b^	Focal hyperautofluorescence^23^	Focal dark green lesions with elevated appearence
NSD^c^	Hypoautofluorescent area^24^	Bright green diffuse area, elevated in appearance
Subretinal deposits	Hyperautofluorescent deposits^4^	Bright green deposits, mostly within the NSD
Pachyvessels	Hyperautofluorescence corresponding to choroidal vessels on MCI^28^	Bright orange vascular network

^a^Retinal pigment epithelium.

^b^Pigment epithelial detachment. ^c^Neurosensory detachment.
2. Imaging features on MCI were determined based on previous descriptions in literature and by correlating a given lesion on OCT^19–22^.
3. RPE atrophy and PED were characterized by number of lesions, area of lesions in millimeter square (mm^2^) and definition of the lesions. NSD was characterized by definition and area of lesion in mm^2^ while subretinal deposits were classified as present or absent. Pachyvessels were graded based on definition alone.
4. Each finding (except subretinal deposits) was defined based on how distinctly its margins were visualized on a given imaging modality using a previously described grading scale^19^. The lesions were hence classified as “margins not visualized”, “barely visible margins” and “clearly visible margins”. The sensitivity and specificity of detecting NSD and subretinal deposits was computed for MCI and FAF. Presence of NSD on SD-OCT and the total number of eyes with subretinal deposits in both investigations (individually and combined) were used as the standards for identifying these findings.
5. RPE atrophy and PED were measured in  a mutually exclusive manner for a given area of measurement made. In case of an area of RPE atrophy within an area of NSD, no deduction in NSD area measurement was made considering the different anatomical location of these lesions. In case of multiple lesions of a given finding (ex: RPE atrophy), the cumulative area of all lesions was considered for analysis and the finding was graded based on the individual lesion with the largest area.
6. For quantitative assessment, the freehand selection tool in ImageJ(NIH, Bethesda, USA) was used to measure area of a given lesion. The fit-spline option was used, once the lesion was measured, to ensure better accuracy.
7. All measurements were made in pixels and were converted to mm^2^ using the scale provided with each image.

### Statistical analysis

Statistical analysis was performed using SPSS version 21 statistical software (IBM corporation, Chicago, Illinois, USA). The means of different characteristics of each finding on MCI and FAF were compared.

Data was first checked for normality using the “descriptive statistics” option on SPSS. The difference between individual data entries (MCI and FAF) for each given subject was calculated and this difference was checked for normality using descriptive statistics. The skewness & kurtosis with their corresponding z-values(range −1.96 to +1.96), the shapiro-wilk’s test (p > 0.05) along with a visual inspection of histograms, Q-Q plots and boxplots were all used to determine normality of data.

Based on these parameters, if the data was parametric, a paired t-test was used to compare the means of characteristics and if the data was non-parametric, the wilcoxon signed rank test was used to compare the means. A p value of < 0.05 was considered to be significant.

## Results

Twenty-five (11 left and 14 right) eyes of twenty-one patients diagnosed with CSCR were included in the study. The study cohort included 19 males and two females. The mean age at presentation was 45.71 ± 10 years and the baseline mean BCVA was logMAR 0.23 (Snellen’s equivalent 20/34). The mean duration of symptoms was 7.01 ± 7.8 months and the most common presenting symptom was blurring of vision.

### Neurosensory detachment

Both MCI and FAF demonstrated the presence of NSD in 13 eyes. The mean cumulative NSD area was statistically higher on MCI compared to FAF(17.2 ± 11.4 vs 15.7 ± 10.7 mm^2^, p = 0.033).Similarly, number of eyes with clearly visible NSD margins (11 vs 7) was higher on MCI in comparison to FAF (Table 2). OCT confirmed the presence of NSD in all 13 eyes translating to 100% sensitivity in detecting NSD for both imaging modalities.

**Table 2:** Comparison of definition (distinctness) of neurosensory detachment (NSD) on multicolour imaging (MCI) and fundus autofluorescence (FAF).

NSD		FAF	
MCI	Margins not visualised	Barely visible margins	Clearly visible margins
Margins not visualised	12	0	0
Barely visible margins	0	1	1
Clearly visible margins	0	5	6

In 19/25 (76%) eyes, the grading was consistent while in six eyes there was a discrepancy with five eyes favouring MCI.

### Subretinal deposits

Subretinal deposits were demonstrated by both investigations in 20 (80%) out of 25 eyes while in three eyes no subretinal deposits were noted in both imaging modalities. Discrepancy was noted in two eyes with sub retinal deposits being demonstrated on MCI alone in one and on FAF alone in the other. Both investigations had a 95.4% sensitivity and 100% specificity in detecting subretinal deposits.

### RPE atrophy

Both MCI and FAF showed RPE atrophic patches in 21(84%) out of 25 eyes. The mean number of lesions (2.4 ± 2.01 vs 2.3 ± 2.08, p = 0.577) and the number of eyes with clearly visible RPE atrophy margins (18 vs 13 - Table 3) was higher on MCI when compared to FAF. MCI also revealed a higher mean cumulative area of RPE atrophy (6.3 ± 6.02 vs 5.7 ± 5.7 mm^2^, p = 0.046).

**Table 3:** Comparison of definition (distinctness) of retinal pigment epithelium (RPE) atrophy on multicolour imaging (MCI) and fundus autofluorecence (FAF).

RPE atrophy		FAF	
MCI	Margins not visualised	Barely visible margins	Clearly visible margins
Margins not visualised	4	0	0
Barely visible margins	0	2	1
Clearly visible margins	0	6	12

In 18/25 (72%) eyes, the grading was consistent while in seven eyes there was a discrepancy with six eyes favouring MCI.

### Pigment epithelial detachments

MCI demonstrated PED in nine eyes in comparison to seven eyes on FAF along with a higher mean number (3.1 ± 3.5 vs 2.2 ± 1.9, p = 0.18) and a higher mean cumulative area (1.3 ± 1.4 vs 1.1 ± 1.2 mm^2^, p = 0.068) of PED. MCI also revealed higher number of eyes with clearly visible PED margins (5 vs 2 - Table 4).

**Table 4:** Comparison of definition (distinctness) of pigment epithelium detachment (PED) on multicolour imaging (MCI) and fundus autofluorescence (FAF).

PED		FAF	
MCI	Margins not visualised	Barely visible margins	Clearly visible margins
Margins not visualised	16	0	0
Barely visible margins	0	2	0
Clearly visible margins	2	3	2

In 20/25 (80%) eyes, the grading was  consistent while in five eyes there was a discrepancy with all five eyes favouring MCI.

### Pachyvessels

MCI showed the presence of pachyvessels in all 25 eyes while FAF demonstrated them in 24 eyes. On FAF, the pachyvessels were consistently defined as “barely visible” in all except two eyes among which one was graded as “clearly visible” and the other as “ no visible” pachyvessels. MCI, on the other hand, revealed clearly visible pachyvessels in all the 25 eyes.

## Discussion

In this study, we compared the characteristics of five different clinical findings on MCI and FAF in CSCR. These characteristics included qualitative (definition) as well as quantitative measures (number of lesions and cumulative lesion area). This study demonstrates the advantages of MCI in comparison to FAF in defining the various clinical findings in CSCR and hence providing us with a better understanding of the disease process. CSCR, depending on the evolution of the disease, presents with a wide gamut of clinical findings. MCI imaging, by means of its ability to penetrate retinal layers differentially and amalgamate the reflectance patterns, is able to pick-up early and subtle clinical changes in patients with CSCR at different anatomical levels. This feature is key in helping us understand and prognosticate the disease in a more comprehensive manner.

**Figure 1:**
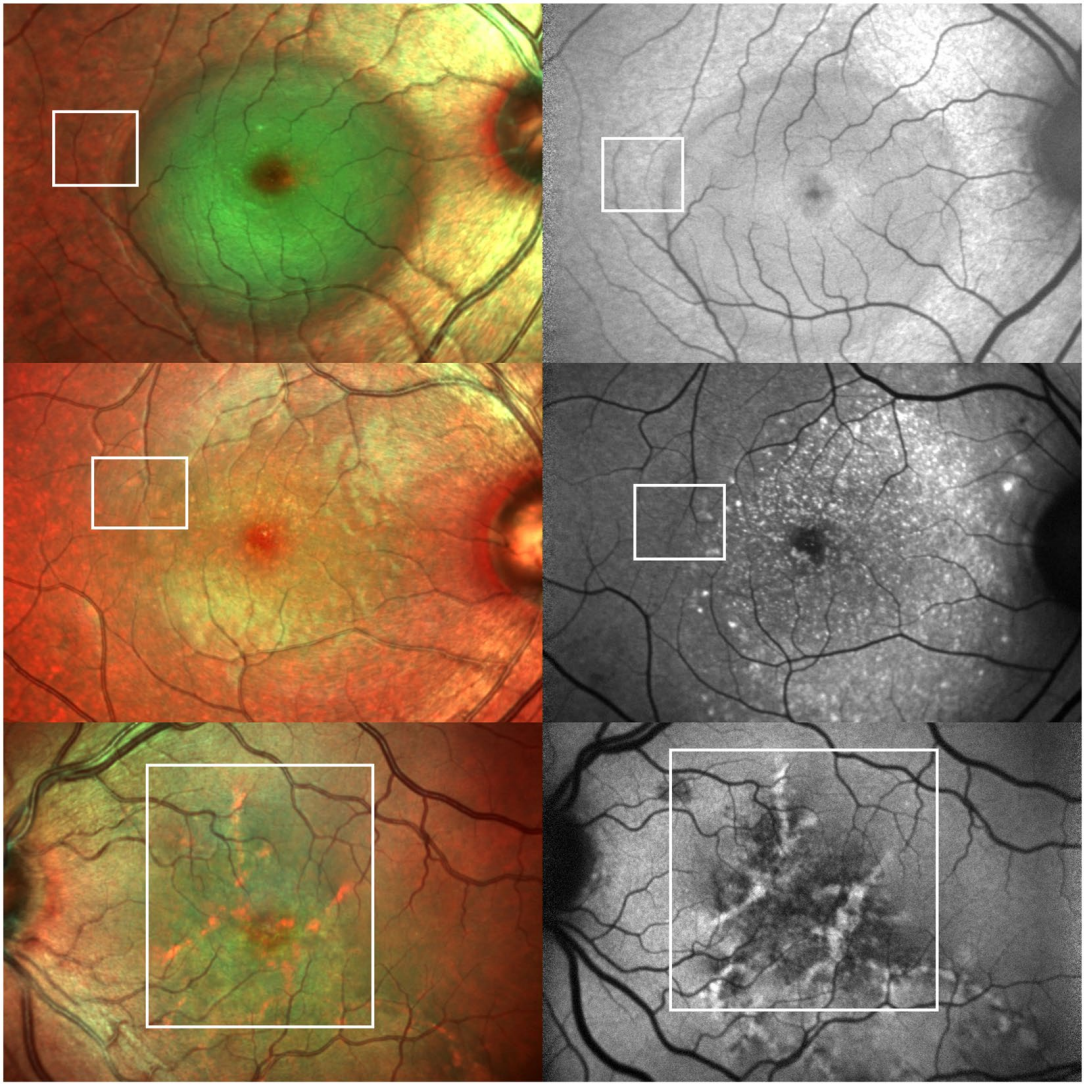
Imaging features of clinical findings in central serous chorioretinopathy (CSCR) on multicolor imaging (MCI) and fundus autofluorescence (FAF) - MCI of various clinical findings are noted on the left while those on the right represent the corresponding FAF images. Top left and right show neurosensory detachment (NSD) with enhanced green reflectance and hypoautofluorescence respectively. Note a few a prominent green subretinal deposits within the NSD in the top left image. Middle left and right demonstrate NSD with subretinal deposits. They appear as discrete green deposits on MCI and as hyperautofluorescent spots on FAF. Bottom left and right depict sequelae of CSCR with a large area of retinal pigment epithelium and inner choroidal atrophy resulting in visualisation of the pachyvessels on both investigations. Note the orange vascular pattern and hyperautofluorescent vessels on MCI and FAF respectively. This is the only eye with clearly visible pachyvessels on FAF. The boxes in all the six images demonstrate colocalisation of pachyvessels on MCI and FAF.

The FAF findings of NSD in CSCR are quite variable and chronicity dependent. NSD in a setting of acute CSCR has been described as hypo (top right Fig. 1) as well as hyper-autofluorescent^23,24^. NSD is represented by a well-defined, green area on MCI owing possibly to altered reflectivity due to the forward shift of the neurosensory retina (top left Fig. 1). The margins of the NSD were consistently better defined on MCI in comparison to FAF (12 vs 8 eyes).

**Figure 2:**
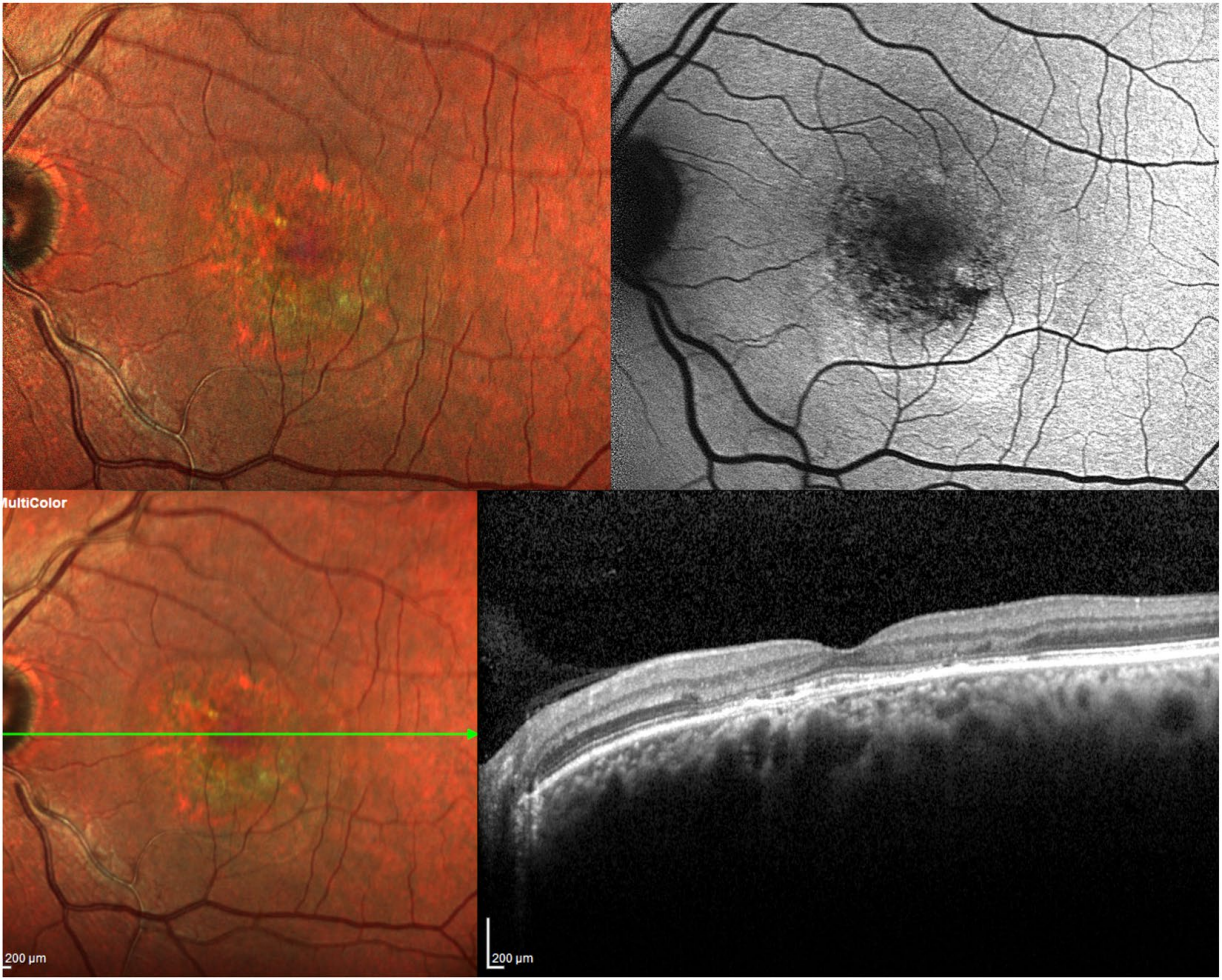
Resolved neurosensory detachment (NSD) on multicolour imaging (MCI) and fundus autofluorescence (FAF) - Left eye MCI (top left) shows an area of altered reflectivity with subretinal deposits and retinal pigment epithelium (RPE) atrophy within while the corresponding FAF (top right) depicts an a ring of hyper-autofluorescence surrounding a inner ring of stippled autofluorescence with a few foci of hypoautofluorescnce. The corresponding spectral domain optical coherence tomography (SD-OCT) at the bottom shows absence of NSD along with outer retinal disruption, altered RPE reflectance and a double-layer sign. This depiction of NSD on MCI and FAF are important in understanding the disease history and the corresponding functional effect.

Considering the high sensitivity of MCI in detecting NSD, it can be used as a reliable adjunct to SD-OCT in detecting NSD. MCI depicts an area of resolved NSD as a zone of altered reflectance with well-defined margins (Fig. 2). These resolved NSD patterns can be employed for correlation with functional imaging modalities such as microperimetry. Based on the above mentioned observations, MCI aids in detecting and following-up NSD in the setting of acute as well as chronic CSCR.

Alten *et al*. evaluated reticular pseudodrusen (RPD) and their target aspect using multimodal imaging, which included MCI and FAF. They described RPD as greenish yellow lesions with yellow cores in case of presence of a target aspect on MCI^20^, Considering the similar anatomical location of RPD and subretinal deposits in CSCR, these deposits displayed a similar reflectance pattern (middle left Fig. 1) These subretinal deposits are represented as granular and punctuate hyperautofluorescent lesions (middle right Fig. 1) on FAF^4,25^. An overlap of the FAF findings with CSCR sequelae in the form of thinning and atrophy of RPE leads altered localization of subretinal deposits on FAF. Moreover hyperautofluorescent deposits exhibit two patterns – smaller,distinct and intense hyperautofluorescent dots which corresponded to the hypererflective subretinal deposits on SD-OCT and larger, ill-defined hyperautofluorescent dots which corresponded to RPE protrusions on SD-OCT^9,26^. This leads to further difficulty in delineating subretinal deposits on FAF.

MCI and FAF were highly specific in identifying subretinal deposits and this translates to better understanding of disease chronicity as both investigations are very efficient in ruling out these deposits. Both investigations failed to detect subretinal deposits in one out of 22 eyes reflecting as 95.4% sensitivity. Considering a very low false negative rate, a combination of both imaging modalities can be considered for better and more comprehensive detection of these deposits.

**Figure 3:**
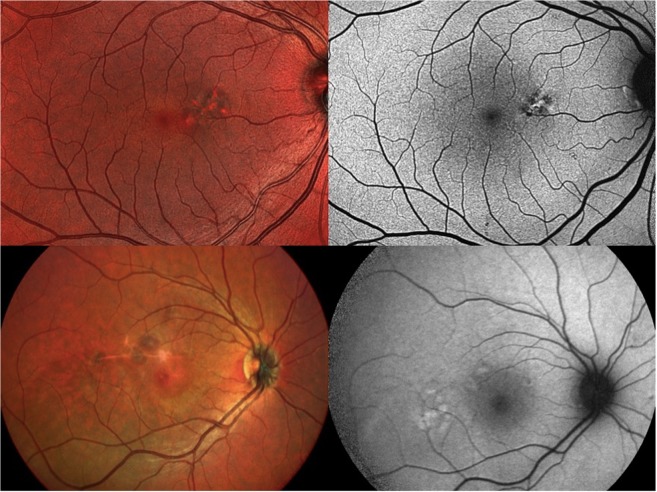
Imaging features of clinical findings in central serous chorioretinopathy (CSCR) on multicolor imaging (MCI) and fundus autofluorescence (FAF) -Clinical findings on MCI (left) and their corresponding FAF images (right) are noted. A discrete patch of retinal pigment epithelium (RPE) atrophy is noted nasal to the macula in the top left and right images which is seen as an area with visible choroidal vasculature surrounded by altered reflectance on MCI and as a hypoautofluorescent area surrounding a central hyperautofluorescene on FAF. Focal dark green lesions noted superior and temporal to macula on MCI (bottom left) and the corresponding areas of focal hyperautofluorescence on FAF (bottom right) depict pigment epithelial detachments.

RPE atrophy, which presents as well defined areas of hypoautofluorescence on FAF^11^, is represented by well-demarcated areas of RPE depigmentation with increased visualization of choroidal vessels on MCI (top right and left- Fig. 3). The choroidal vessels appear as a bright orange vascular network, which was akin to the description by Moussa NB *et al*.^21^. They looked at width and area of geographic atrophy(GA) secondary to age-related macular degeneration on multiple imaging modalities (colour fundus photo, multicolor imaging, FAF and spectral domain – optical coherence tomography) and concluded that MCI is a valuable tool in assessing GA. The higher infrared wavelength (820 nm) of MCI in comparison to the blue peak autofluorescence (488 nm) gives MCI a distinct edge in imaging changes at the level of the RPE. A near-infrared FAF would correlate better with MCI considering the input wavelengths.

PED has been typically defined as a focal hyper-autofluorescent area of FAF (bottom right Fig. 3). Von ruckmann *et al*. described PED on  FAF as an area of mild hyperautofluorescence surrounded by a ring of hypoautofluorescence attributing the dark ring to attenuation of the exciting light source^24^. A previous review on MCI depicted PED as dark greenish lesion with well-defined borders^1^. In our cohort of CSCR patients, PED appeared similar to the above mentioned description on MCI with a higher detection rate and better definition compared to FAF(bottom left Fig. 3). The reflectance patterns of an NSD and PED differ based on the elevated tissue with the retinal layers reflecting more at shorter wavelengths (450–540 nm) and melanin (the main constituent of pigment granules in RPE) progressively reflecting at longer wavelengths^22,27^.

Choroidal vessels have been visualized as hyperautofluorescent linear lines on short-wavelength FAF in studies in the past. This FAF finding has been typically described in conditions (choroidal sclerosis and high myopia) wherein the RPE and choriocapillaris have undergone thinning and hence led to enhanced visualization of underlying larger choroidal vessels^28,29^. We observed similar findings in one of our subjects wherein diffuse RPE and choriocapillaris atrophy secondary to CSCR lead to clearly visible hyperautofluorescent pachyvessels on FAF (bottom right Fig. 1). The rest of the eyes showed barely visible pachyvessels on FAF due to differential RPE and inner choroidal atrophy.On MCI, the pachyvessels appeared to be orangish in colour and arranged in the form of a vascular network (bottom left Fig. 1). Considering the similarity in description, it is very challenging to differentiate pachyvessels from RPE atrophy on MCI without referring to the corresponding FAF images. The presence of variable degrees of RPE atrophy aids us in visualising the underlying pachyvessels on MCI in CSCR.

The description of pachyvessels on MCI in our study differs from that of Muftuoglu *et al*. who described choroidal vessels in a normal retina to be green in reflectance^22^. Considering the twin absorption peaks (413 and 540 nm) of oxyhemoglobin and its predominance in choroidal blood flow, the orangish appearance of the choroidal vessels is better understood^27^.

The main drawbacks of our study include its retrospective nature and a limited sample size. Use of multimodal imaging including blue reflectance, red-free images, infrared reflectance^30^, SD-OCT^31^, OCTA and angiography (fluorescein and indocyanine green)^16–18^ would have aided in better characterisation of clinical findings on MCI as they would have helped in identifying and colocalising the more subtle clinical findings.The use of en-face imaging^32^ and volumetric SD-OCT data helps in more accurate anatomical localization of clinical findings and this would further enhance our understanding of how changes in CSCR appear on multicolor imaging.

To conclude, MCI was found to be an excellent tool in detecting and quantifying the various characteristic clinical findings in CSCR. The ability to detect early and subtle changes along with a consistent edge over FAF in lesion characterization makes MCI a frontrunner for routine use in the identification and follow-up of patients with CSCR.

## Data Availability

The datasets generated during and/or analysed during the current study are included in this published article and further information regarding the same is available from the corresponding author on reasonable request.
